# Help-Seeking for Sexual Difficulties and the Potential Role of Interactive Digital Interventions: Findings From the Third British National Survey of Sexual Attitudes and Lifestyles

**DOI:** 10.1080/00224499.2019.1586820

**Published:** 2019-03-25

**Authors:** Lorna J. Hobbs, Kirstin R. Mitchell, Cynthia A. Graham, Viktoriya Trifonova, Julia Bailey, Elizabeth Murray, Phil Prah, Catherine H. Mercer

**Affiliations:** 1 eHealth Unit, Research Department of Primary Care and Population Health, University College London; 2 Institute of Health and Wellbeing, University of Glasgow; 3 Department of Psychology, University of Southampton; 4 Institute for Global Health, University College London

## Abstract

Sexual difficulties are common and can negatively impact health and well-being. A wide range of support is available, but there are multiple barriers to accessing help. Interactive digital interventions (IDIs) for sexual difficulties have the potential to provide a convenient, wide-reaching, and cost-effective source of support, but little is known about who might use them. We explored the potential reach of IDIs by assessing the prevalence of help-seeking among people with distressing sexual difficulties, including who seeks which sources of help. Data came from sexually active men and women, ages 16 to 74, participating in Britain’s third National Survey of Sexual Attitudes and Lifestyles (Natsal-3) (N = 11,637). Help/advice was sought by less than half of those with distressing sexual difficulties, and help-seeking was associated with younger age in women but not men. The most popular sources of support were family doctor (47.5% to 54.8%), Internet (22.0% to 25.6%), and family/friend (20.7% to 41.8%), with older participants (≥ 35), particularly men, preferring to seek help from a family doctor, and younger participants (<35) preferring to seek help from the Internet or family/friend. Despite a paucity of good digital support sites for sexual function, the Internet is a common source of help. As Internet access continues to increase, so too does the potential for well-designed IDIs to support those with sexual difficulties.

Sexual function is a key component of health and well-being (Bancroft, Loftus, & Long, 2003; Connor et al., 2011; Glasier, Gulmezoglu, Schmid, Moreno, & Van Look, 2006; Jern, Gunst, Sandnabba, & Santtila, 2012; Patrick et al., 2013). Sexual difficulties are common in Britain, with an estimated 51% of women and 42% of men reporting one or more sexual difficulties for three months or longer in the past year in Britain’s most recent National Survey of Sexual Attitudes and Lifestyles (Natsal-3; Mitchell et al., 2013). In terms of more serious problems, 4.2% of men and 3.6% of women in the same study reported problems that approximated the *Diagnostic and Statistical Manual of Mental Health Disorders*, Fifth Edition (*DSM*-5; American Psychiatric Association, 2013), criteria for sexual dysfunction (Mitchell et al., 2016). One of these criteria, distress, is typically used to differentiate mild and transient difficulties from more severe problems and provides a means of identifying people who may seek help/advice.

Many sexual difficulties are amenable to psychological treatment (Frühauf, Gerger, Schmidt, Munder, & Barth, 2013), and education and reassurance is often all that is needed. Yet there are multiple individual and systemic barriers to accessing face-to-face help, including not knowing where to seek help, a severe lack of specialty services, long waiting lists for existing specialty services, high costs for private services, and discomfort talking about sex with health professionals (Adegunloye & Ezeoke, 2011; Akre, Michaud, & Suris, 2010; British Psychological Society (BPS), 2006; Hinchliff, Gott, & Wylie, 2009; Medical Foundation for HIV and Sexual Health (MEDFASH), 2008). Family doctors are the most common source of support in the United Kingdom (Mitchell et al., 2013), but they often lack time and expertise.

In the past decade or so, there has been a global increase in online help-seeking for different health issues and a proliferation of online sources of health-related information and support (Bailey et al., 2015; Currie & Seddon, 2014; Wachter, 2016). There are numerous international policy commitments to the integration of digital technologies in health care systems (Department of Health, 2012a, 2012b, 2014; Department of Health and Human Services, 2011, 2015; European Commission, 2018) to meet health care’s “triple aim” of better care, better outcomes, and less cost (Berwick, Nolan, & Whittington, 2008). This is particularly key given that health care budgets are severely constrained in most countries.

Interactive digital interventions (IDIs) that enable help/advice to be provided online could provide a potentially cost-effective alternative to face-to-face help (Portnoy, Scott-Sheldon, Johnson, & Carey, 2008; Ritterband et al., 2011; Ritterband & Tate, 2009). IDIs have found to be effective in many different areas of health/mental health (Bailey et al., 2010; Barak, Hen, Boniel-Nissim, & Shapira, 2008; Brown et al., 2012; Khadjesari, Murray, Hewitt, Hartley, & Godfrey, 2011; Noar, Pierce, & Black, 2010) and may be particularly well suited to the treatment of sexual difficulties as they provide innovative ways to access hard-to-reach populations (Boeltzig & Pilling, 2007). The privacy and anonymity of IDIs are particularly appealing given the stigma and embarrassment often associated with sexual difficulties (Thomas, McLeod, Jones, & Abbott, 2014). In addition, qualitative findings indicate that people are attracted to the convenience, accessibility, tailoring, and self-pacing of IDIs (Hobbs, 2016). People with sexual difficulties who used a program of online sex therapy reported many beneficial effects, including reduced embarrassment and shame, increased understanding of their sexual difficulties, increased sexual confidence, and greater perceived ability to manage the difficulty or speak to a health professional about difficulties (Hobbs, 2016). The Internet offers a potentially cost-effective platform from which to widely disseminate psychotherapeutic interventions, and IDIs have real potential to address some of the need for help in this area (Bailey et al., 2015).

Emerging evidence suggests that IDIs can have a positive impact on sexual function. For example, a meta-analysis estimated a standardized mean difference of 1.05 (95% confidence interval [CI] = 0.14 to 3.05) between those with sexual function problems who used an IDI compared to those who received wait-list or standard care (Hobbs, 2016), which is a large effect size using Cohen’s criteria (Cohen, 1988; Higgins & Green, 2011). However, little is known about the potential reach of IDIs and the popularity of the Internet relative to other sources of help (Bennett & Glasgow, 2009).

The aim of this study was to estimate the extent of unmet need for help with sexual difficulties and to explore the potential of the Internet as a mode of delivery of help. To do so we used nationally representative data to estimate the prevalence of seeking help/advice for one’s sex life in the past year in people resident in Britain who reported distressing sexual difficulties. We sought to examine the prevalence of use of different sources of help and to identify sociodemographic characteristics of those seeking help from the Internet to inform the design and delivery of future IDIs.

## Method

### Participants

Natsal-3 is a stratified probability sample survey of 15,162 men and women aged 16 to 74 years resident in Britain who were interviewed between September 2010 and August 2012.

### Procedure

The survey used a multistage, clustered, and stratified probability sample design, with post-code sectors as the primary sampling units (PSUs). Trained interviewers conducted a computer-assisted personal interview (CAPI), including a nested computer-assisted self-interview (CASI). The estimated survey response rate was 57.7% (Mercer et al., 2013). After weighting to adjust for unequal probabilities of selection, the Natsal-3 sample was broadly representative of the British population according to the 2011 Census (Erens et al., 2013). Full details of the survey methods of Natsal-3 are published elsewhere (Erens et al., 2013; Mercer et al., 2013). The Oxfordshire Research Ethics Committee A (10/H0604/27) approved the Natsal-3 study, and participants provided informed consent orally (Mercer et al., 2013).

### Measures

#### Distressing Sexual Difficulties

These were selected items from a validated measure of sexual function designed for the survey (Mitchell et al., 2013; Mitchell, Ploubidis, Datta, & Wellings, 2012). In the CASI, sexually active participants, defined as those reporting one or more sexual partners (of either gender) in the past year (*N* = 11,637; 42% or 4,901 of which were men) were asked if they had experienced any of the following for three months or longer in the past year: (1) lacked interest in having sex; (2) lacked enjoyment in sex; (3) felt anxious during sex; (4) felt physical pain as a result of sex; (5) felt no excitement or arousal during sex; (6) did not reach a climax (experience an orgasm) or took a long time to reach a climax despite feeling excited/aroused; (7) reached a climax (experienced an orgasm) more quickly than you would have liked; and (8) had an uncomfortably dry vagina (asked of women only); or (9) had trouble getting or keeping an erection (asked of men only). If they reported one or more of these problems, they were then asked how long had they experienced this/these, and then whether they felt distressed about this (answer options: *Not at all distressed, A little distressed, Fairly distressed, Very distressed*). For this article, we labeled participants who reported at least one sexual function problem that they were fairly or very distressed about as having “distressing sexual difficulties.”

#### Sources of Help/Advice

In the CASI, all sexually active participants were then asked: “Have you sought help or advice regarding your sex life from any of the following sources in the past year?” Answer options: (1) *Family member/friend*; (2) *Information and support sites on the Internet*; (3) *Self-help books/information leaflets*; (4) *Self-help groups*; (5) *Helpline*; (6) *GP (general practitioner)/family doctor*; (7) *Sexual health/GUM/STI clinic*; (8) *Psychiatrist or psychologist*; (9) *Relationship counselor;* (10) *Other type of clinic or doctor*; (11) *Have not sought any help*. Participants could report more than one option. Those reporting at least one of responses 1 through 10 were labeled help-seekers; those who reported “information and support sites on the Internet” were labeled Internet help-seekers.

#### Sociodemographics

We focused on age, sexual identity (as measured by the Office for National Statistics [ONS, 2009]), relationship status, a summary measure of academic qualifications, social class (as measured by the National Statistics Socio-Economic Classification [NS-SEC]), and area-level deprivation (as measured by quintiles of the Index of Multiple Deprivation [IMD]; Payne & Abel, 2012). See Table 1 for the response options for these variables. These variables were chosen as they are largely stable concepts that could help predict the reach of IDIs and thus help inform the design and delivery of future IDIs for sexual difficulties and modification of existing ones (e.g., via tailoring dimensions, choice of content and design features, language style, and navigational architecture).

**Table 1.** Variations in the Prevalence of Reporting Seeking Help/Advice for One’s Sex Life, by Gender, Among Sexually Active Participants Reporting One or More Distressing Sexual Difficulties

	Women				Men			
	Prevalence (%) of Reporting Seeking Help/Advice (95% CI)	Odds Ratio (95% CI)	Denominator (Weighted, Unweighted)		Prevalence (%) of Reporting Seeking Help/Advice (95% CI)	Odds Ratio (95% CI)	Denominator (Weighted, Unweighted)	
All	40.18% (36.35–44.13)		718	858	41.12% (36.32–46.10)		625	524
Age		*p *= 0.008				*p *= 0.954		
16–24	49.18% (42.30–56.09)	1.00	145	256	38.72% (29.17–49.22)	1.00	93	125
25–34	46.71% (40.24–53.28)	0.91 (0.63– 1.31)	174	300	38.99% (30.25–48.48)	1.01 (0.57–1.81)	125	147
35–44	35.78% (26.47–46.31)	0.58 (0.34–0.98)	117	97	39.81% (28.26–52.61)	1.05 (0.53–2.07)	110	73
45–54	30.37% (21.94–40.37)	0.45 (0.27–0.77)	154	111	43.89% (31.58–57.00)	1.24 (0.63–2.44)	123	67
55–64	42.54% (30.78–55.22)	0.77 (0.43–1.36)	95	70	45.19% (33.43–57.50)	1.30 (0.67–2.54)	119	74
65–74	20.84% (8.60–42.42)	0.27 (0.10–0.77)	33	24	37.67% (23.62–54.15)	0.96 (0.43–2.12)	56	38
Sexual identity		*p *= 0.741				*p *= 0.944		
Heterosexual	40.05% (36.11–44.12)	1.00	688	816	41.08% (36.05–46.29)	1.00	586	487
Nonheterosexual	43.11% (26.85–61.01)	1.13 (0.54–2.40)	30	42	41.75% (25.47–60.06)	1.03 (0.47–2.24)	39	37
Relationship status		*p *= 0.031				*p *= 0.551		
Married/civil partnership	34.73% (29.21–40.70)	1.00	379	317	37.33% (29.97–45.32)	1.00	315	180
Living with a partner but not as married/civil partnership	44.21% (35.99–52.76)	1.49 (0.97–2.28)	133	180	45.91% (34.60–57.7)	1.43 (0.80–2.53)	107	84
Steady relationship but not cohabiting	47.85% (39.36–56.46)	1.72 (1.13–2.63)	113	194	44.57% (35.04–54.53)	1.35 (0.80–2.28)	96	123
Not in a steady relationship	47.27% (39.03–55.66)	1.68 (1.09–2.61)	93	167	43.44% (34.74–52.55)	1.29 (0.79–2.11)	106	134
Academic qualifications		*p *= 0.024				*p *= 0.873		
Left school having passed exams	41.92% (37.75–46.21)	1.00	605	738	40.79% (35.63–46.17)	1.00	514	437
Left school at or before, 16 without passing exams	28.46% (19.60–39.36)	0.55 (0.33–0.93)	96	95	39.63% (27.39–53.34)	0.95 (0.53–1.72)	96	70
National Statistics Socio-Economic Classification		*p *= 0.115				*p *= 0.302		
Managerial/professional	42.80% (36.01–49.87)	1.00	254	260	43.98% (35.79–52.51)	1.00	233	168
Intermediate occupation	36.18% (27.73–45.58)	0.76 (0.46–1.24)	140	167	47.15% (35.50–59.13)	1.14 (0.63–2.04)	100	80
Semiroutine/routine occupation	38.06% (31.54–45.04)	0.82 (0.55–1.23)	196	251	34.45% (27.07–42.67)	0.67 (0.41–1.09)	211	194
No job	31.71% (21.00–44.79)	0.62 (0.33–1.16)	68	79	33.90% (16.99–56.25)	0.65 (0.25–1.73)	36	27
In full-time education	52.88% (40.88–64.54)	1.50 (0.86–2.62)	58	97	46.07% (30.67–62.26)	1.09 (0.52–2.30)	42	53
Quintile of Index of Multiple Deprivation		*p *= 0.502				*p *= 0.464		
1 (Least deprived)	44.06% (36.13–52.32)		1.00	161	147	39.55% (29.80–50.22)	139	108
2	40.11% (30.64–50.37)	0.85 (0.50–1.44)	135	143	38.55% (26.90–51.67)	0.96 (0.48–1.91)	112	84
3	43.63% (35.33–52.30)	0.98 (0.61–1.58)	154	175	37.04% (27.99–47.10)	0.90 (0.50–1.63)	132	111
4	36.85% (29.76–44.55)	0.74 (0.47–1.17)	157	202	50.61% (39.42–61.75)	1.57 (0.83–2.94)	114	102
5 (Most deprived)	35.61% (28.16–43.83)	0.70 (0.43–1.13)	125	177	40.83% (30.72–51.79)	1.05 (0.57–1.96)	129	119

*Note*. The associations between help-seeking and relationship status and education level in women were no longer statistically significant after adjusting for age.

### Data Analysis

Data were analyzed using the complex survey data analysis function in STATA Version 12.1, which accounts for stratification, clustering, and weighting of data. The denominator was initially sexually active participants to show the prevalence of reporting one or more distressing sexual difficulties in the past year— the target population. Then, to demonstrate the potential unmet need for help/advice, we calculated the prevalence (%, 95% confidence interval [CI]) of help-seeking among those sexually active participants who reported any distressing sexual difficulty/difficulties and considered whether this varied by key sociodemographic variables. The chi-square statistic was used to test for statistically significant differences with alpha set at 0.05 for all analyses. We also calculated odds ratios (ORs) and age-adjusted odds ratios (aORs), given the strong association between age and the factors considered. We do not present further aORs partly because of statistical power issues and also so as not to “explain away” associations with help-seeking. These analyses are presented separately by gender due to differences in the experiences and reporting of sexual behaviors (Mercer et al., 2013), as well as the sexual scripts which shape these behaviors (Mitchell et al., 2011; Simon & Gagnon, 2003).

For each of the sources of help/advice, we ascertained how commonly these were reported among those who reported distressing sexual difficulties and that they had sought help. These percentages were again calculated separately for women and men, and also for two age groups: younger participants (those under age 35) and older participants (those age 35 and older). This age cutoff was chosen as it is likely to broadly tally with an age at which many people are likely to be in long-term relationships and have children, both of which are known to correlate with sexual function difficulties (Mercer et al., 2013; ONS, 2018; Wellings et al., 2013). We used percentages to describe the sociodemographic profile of participants who reported distressing sexual difficulties and had used the Internet for help/advice and presented them by gender. Patterns in the data were commented on, but significance testing was not performed because the cell numbers were too small. Item nonresponse in Natsal-3 was low—below 0.5% in the CAPI and 1% to 3% in the CASI (Erens et al., 2013)—and therefore participants with missing data on a particular variable were excluded from the analysis.

## Results

Among the sexually active participants, 10.3% (95% CI: 9.4% to 11.4%) of men (unweighted: 524/4,901) and 12.5% (95% CI: 11.6% to 13.4%) of women (unweighted: 858/6,736) reported at least one distressing sexual function problem (*p *= 0.003 for gender difference). Of these participants, 41.1% (95% CI: 36.3% to 46.1%) of men and 40.2% (95% CI: 36.4% to 44.1%) of women reported seeking help/advice for their sex life in the past year (help-seeking, *p* = 0.770 for gender difference) (Table 1).

**Figure 1.**
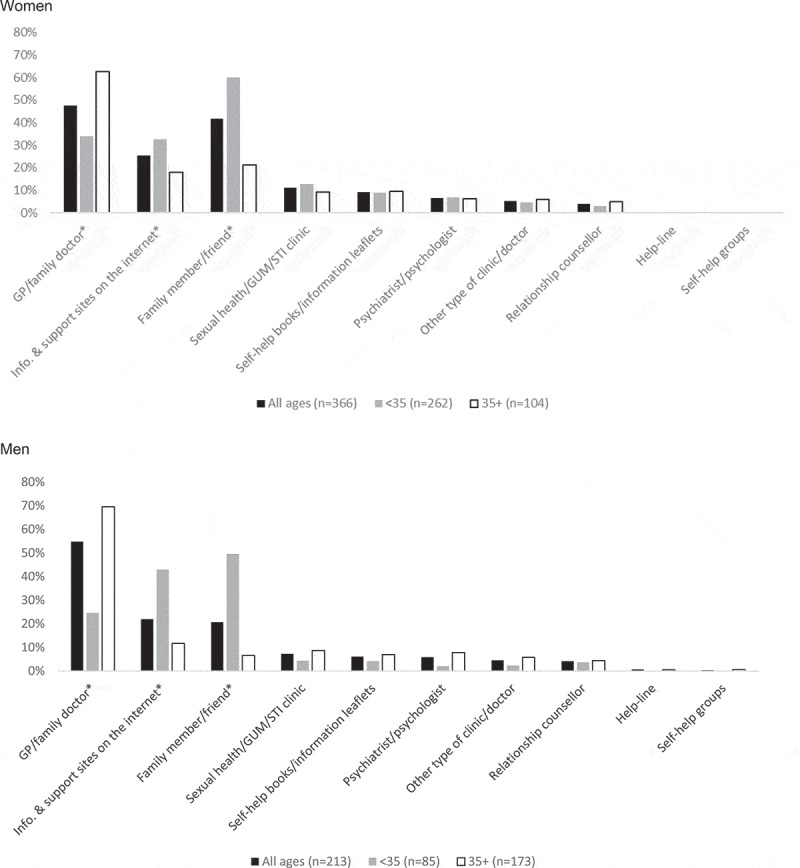
Sources of help/advice used by participants who reported distressing sexual difficulties who sought help/advice for their sex lives, by gender and age group; *denotes a statistically significant difference by age-group (under age 35 versus age 35 and older).

The prevalence of help-seeking declined with age among women: Approximately half of women aged 16 to 24 reporting distressing sexual difficulties had sought help, but this dropped to around one in five of women aged 65 to 74 (Table 1). No association with age was observed for men. Help-seeking was also more common among women—but not men—in nonmarital relationships and among those with higher levels of education, although these associations were no longer significant after adjusting for age (data not shown). No differences in help-seeking were observed for either gender according to measures of social class, area-level deprivation, or sexual identity. The prevalence of different sources of help/advice sought by the 366 women and 213 men who reported distressing sexual difficulties and help-seeking is shown in Figure 1.

The most commonly reported source was a family doctor, reported by around half of women and men with distressing sexual difficulties (47.5%, 95% CI: 41.2% to 53.9%; 54.8%, 95% CI: 47.0% to 62.5%; respectively). For women, talking to a family member or friend (41.8%, 95% CI: 35.8% to 48.2%) was the second most commonly reported source, followed by seeking information and support via the Internet (25.6%, 95% CI: 21.0% to 30.9%). For men, the second most common source of help was the Internet (22.0%, 95% CI: 16.6% to 28.6%), followed by talking to a family member or friend (20.7%, 95% CI: 15.5% to 27.2%). There were clear differences with age. While participants age 35 and older most commonly reported their family doctor, those under 35 were much more likely to report a family friend or the Internet, and this trend was particularly evident among men.

Table 2 shows the sociodemographic profile of the 98 women and 57 men who reported distressing sexual difficulties and who had sought help/advice online. These participants were generally young (around two-thirds were under age 35), with varied relationship status, and a sizable minority reporting nonheterosexual identity (higher than in general population; Mercer et al., 2013). Around one-third of women and half of men had managerial/professional socioeconomic status, and participants lived in areas with varying levels of deprivation.

**Table 2.** The Sociodemographic Profile of Participants Who Reported Distressing Sexual Difficulties and Seeking Help/Advice for Their Sex Life via the Internet, by Gender

Sociodemographics	Women		Men	
	Unweighted, Weighted Denominators: 98,74		Unweighted, Weighted Denominators: 57,57	
	% (*n*)	[95% CI]	% (*n*)	[95% CI]
Age				
16–24	30.63 (39)	[21.86%–41.08%]	32.30 (21)	[20.51%–46.86%]
25–34	36.50 (41)	[26.50%–47.82%]	32.08 (22)	[20.53%–46.35%]
35–44	11.40 (7)	[5.54%–22.01%]	14.04 (5)	[5.66%–30.78%]
45–54	10.05 (6)	[4.25%–21.94%]	4.82 (2)	[1.12%–18.44%]
55–64	11.42 (5)	[4.84%–24.65%]	11.87 (5)	[4.70%–26.90%]
65–74	0	—	4.89 (2)	[1.22%–17.62%]
Sexual identity				
Heterosexual/straight	93.94 (91)	[86.90%–97.31%]	89.81 (52)	[76.06%–96.07%]
Other	6.06 (7)	[2.69%–13.10%]	10.19 (5)	[3.93%–23.94%]
Relationship status at interview				
Married/civil partnership	39.68 (27)	[28.53%–52.01%]	28.55 (11)	[16.17%–45.29%]
Living with partner but not married/civil partner	19.12 (18)	[11.83%–29.39%]	16.41 (8)	[7.95%–30.83%]
Steady relationship but not cohabiting	26.82 (32)	[18.34%–37.43%]	34.10 (22)	[21.80%–49.98%]
Not in a steady relationship	14.38 (21)	[8.84%–22.53%]	21.95 (14)	[11.80%–34.42%]
Academic qualifications				
Left school having passed exams	96.70 (93)	[84.31%–99.38%]	97.25 (52)	[88.36%–99.40%]
Left school ≤ 16, without passing exams	3.30 (2)	[0.62%–15.69%]	2.75 (2)	[0.60%–11.64%]
National Statistics Socio-Economic Classification				
Managerial/professional	34.93 (33)	[24.82%–46.61%]	49.17 (26)	[34.77%–63.71%]
Intermediate	18.62 (17)	[10.72%–30.37%]	7.08 (3)	[2.04%–21.76%]
Semiroutine/routine	21.13 (20)	[13.40%–31.68%]	19.81 (13)	[10.97%–33.13%]
No job currently	8.73 (6)	[3.41%–20.60%]	1.71 (1)	[0.24%–11.28%]
Student	16.59 (22)	[10.44%–25.32%]	22.23 (14)	[12.54%–36.31%]
Quintile of Index of Multiple Deprivation				
Least deprived (1)	25.25 (19)	[16.04%–37.39%]	21.56 (11)	[11.67%–36.38%]
2	13.25 (14)	[7.17%–23.20%]	11.88 (8)	[5.69%–23.14%]
3	24.70 (24)	[15.79%–36.45%]	20.59 (10)	[10.91%–35.43%]
4	18.69 (19)	[11.75%–28.41%]	34.31 (18)	[21.39%–50.06%]
Most deprived (5)	18.11 (22)	[11.59%–27.18%]	11.66 (10)	[5.81%–22.03%]

## Discussion

In this large, national probability survey of the British population aged 16 to 74 years, fewer than half of women and men with distressing sexual difficulties in the past year reported that they had sought help/advice for their sex lives during this time. This leaves the majority of those with a distressing sexual difficulty reporting that they had not sought help, demonstrating a large unmet need (Mitchell et al., 2016). Overall, there were few sociodemographic differences between those who did and did not report seeking help, particularly after taking account of age. Women (but not men) help-seekers were generally younger. Participants (women and men) who sought help online tended to be younger and come from a range of socioeconomic backgrounds (as measured by educational attainment and profession type).

The top three sources of help/advice reported by those men and women who had sought help were a general practitioner/family doctor, family and friends, and the Internet (order varying by age), which is consistent with findings in other national surveys of self-reported sexual difficulties (Laumann, Paik, & Rosen, 1999; Moreira et al., 2005; Moreira, Glasser, Nicolosi, Duarte, & Gingell, 2008). While the level of Internet help-seeking was relatively low, at around one in 10 men and women, this is likely to underestimate current levels of online help-seeking because Internet access and smartphone ownership continue to increase annually (ONS, 2017), as does the number of help sources available online (Bailey et al., 2015).

### Comparison of Findings With Previous Research

The estimate that 10.3% of men and 12.5% of women reported at least one distressing sexual difficulty is of similar magnitude to those reported by other studies that have taken account of distress (Shifren, Monz, Russo, Segreti, & Johannes, 2008). The finding that, of those who reported one or more distressing sexual difficulty, 41.1% of men and 40.2% of women sought help and advice for their sex lives is also consistent with previous estimates across different cultures (ranging from 42% to 76% in men and 33% to 68% in women; Laumann, Glasser, Neves, & Moreira, 2009; Moreira et al., 2008). Men are typically less likely to seek help for general health-related issues (Galdas, Cheater, & Marshall, 2005; Koydemir-Özdena & Erelb, 2010), but this does not seem to be true for sexual difficulties (Moreira et al., 2008). We found that younger women were more likely than older women to seek help for sexual difficulties (with no association with age for men), which was not consistent with previous research (Laumann et al., 2009; Moreira et al., 2008). However, it is likely that this association was missed by previous studies (Laumann et al., 2009; Moreira et al., 2008) where the age range (i.e., over 40 years old) was much narrower than the age range in Natsal-3. It is important that any future assessments of real-world effectiveness of IDIs for sexual difficulties take this association into account. The lack of association between educational attainment and socioeconomic status after adjusting for age and help-seeking was a similar finding to previous studies (Moreira et al., 2005) and suggests that, unlike many other areas of health, there is no socioeconomic divide in seeking help for sexual difficulties. Although the data cannot explain this discrepancy, it could be that sexual shame and the taboo associated with talking about sex (Clark, 2017; Shadbolt, 2009) transcends socioeconomic boundaries, but this suggestion is speculative. Sexual shame is thought to be culturally, socially, and politically shaped, and directly constructed and experienced through education, history, religious traditions, media/social media, and relationships (i.e., familial relationships, but also through professional relationships with doctors, nurses, teachers, and so on; Shadbolt, 2009). Current evidence has failed to demonstrate any one group being more susceptible to sexual shame than another group, but this is an underresearched area (Clark, 2017).

The three most popular sources of help-seeking for sexual difficulties in Britain were similar to those found in previous studies across a range of different countries (Moreira et al., 2005, 2008). Preferences differed according to age, with age-35-and-over participants preferring to seek help from a general practitioner/family doctor and under-age-35 participants preferring to seek help from family/friends. This finding may reflect the types of sexual difficulties that people tend to experience at different ages (Mitchell et al., 2016), as well as younger generations being more comfortable accessing information from the Internet.

The digital divide in Internet use found in the wider population (ONS, 2017), and more specifically with regard to online health consumption (Kummervold et al., 2008; Wangberg et al., 2008), was largely present in our data, with people who had sought help/advice for their sex lives online being young, educated, and of managerial/professional socioeconomic status. A digital divide has the potential to worsen health inequities, and therefore IDIs must be designed and delivered in such a way that they are maximally accessible and relevant to people of all backgrounds.

### Strengths and Limitations

Using data from a large national probability survey, the Natsal-3, enabled us to generate findings on help-seeking among people reporting distressing sexual difficulties that are generalizable to the British population. We considered key sociodemographic variables that represent potential targets for the design and marketing of IDIs. Natsal-3’s survey response rate (57.7%) was similar to other large social surveys conducted in Britain (Craig & Mindell, 2011; Park, Clery, Curtice, Phillips, & Utting, 2012), and the use of the CASI likely reduced interviewer bias and item nonresponse/missing data.

A limitation of this secondary data analysis was that the item on help-seeking was not specific to sexual difficulties but rather asked about help/advice for one’s “sex life.” Thus, even among those with distressing sexual difficulties, we cannot conclude that the help they sought specifically related to those difficulties. However, in the questionnaire, the help-seeking question came immediately after the questions about sexual difficulties, so it is reasonable to assume that many of the participants were thinking about sexual difficulties when answering the help-seeking question. It was also not possible to determine the degree of support people obtained online (e.g., from obtaining basic information to taking part in a course of online therapy). It was also not possible to examine other variables, such as cultural factors or health beliefs, that may play a significant role in determining help-seeking behavior, for example, beliefs around whether someone should or should not seek help, and from whom, especially for such a sensitive area as sexual difficulties (Laumann et al., 2009; Moreira et al., 2005, 2008). The help-seeking question did provide a measure of actual (albeit self-reported) behavior, rather than intention to engage in a particular behavior, and therefore avoided the intention–behavior gap problem found in many other survey studies investigating behavior (Sheeran, 2002).

Numbers were insufficient to observe how help-seeking varied by sexual difficulty.Thus, we combined participants regardless of difficulty, resulting in a heterogeneous group. Due to the small number of non-White participants who reported a distressing sexual difficulty, we were not able to look at variations in help-seeking by ethnicity. Finally, due to small numbers we were unable to statistically explore how Internet help-seeking varied by individual characteristics beyond simple descriptive statistics.

### Implications

The relatively low level of help-seeking in people with distressing sexual difficulties indicates a significant unmet need in Britain. International interest in digital health and the changing climate of health service delivery globally suggest there is an emerging role for IDIs for sexual difficulties. It is also important to invest in service delivery methods that are resilient to funding cuts. In the context of poorly resourced and scarce professional services, IDIs delivered online could provide a convenient, scalable, and potentially cost-effective alternative to face-to-face therapy (Hobbs, 2016). IDIs could be offered in conjunction with existing systems, such as electronic appointment bookings, reminders, and/or online sexual health services.

IDIs for sexual difficulties could be relevant for almost one in 10 of people aged 16 to 74 living in Britain (10.5% of men and 12.5% of women) who report distressing sexual difficulties. A more conservative estimate of the potential market for IDIs may be to consider those who have actually sought help/advice, estimated as 40.6% of the sexually active population with distressing problems, and thus around 4% of the British population aged 16 to 74, which amounts to more than 2.2 million people. These estimates of the size of the potential IDI market assume that going online for help/advice for sexual difficulties is acceptable, and research suggests that this is likely to be the case (Hobbs, 2016).

IDIs for sexual difficulties are attractive because they help reduce some of the many barriers to accessing face-to-face help (e.g., discomfort talking about sex with health professionals, a lack of clinician time, a severe lack of specialty services, limited funding and long waiting lists for existing specialty services, and high costs of private services). Although some people prefer face-to-face contact with a professional, IDIs have been found to meet many users’ needs in terms of features, usability, and perceived outcomes (Hobbs, 2016). The development of IDIs in this area has the potential to reach an even greater number of people, including those who have not yet sought help but might benefit from it (Mitchell et al., 2016).

### Unanswered Questions and Future Research

More research is needed to explore the particular aspects of people’s sex lives that they find distressing and the types of help they have sought (or would seek) to provide a more sensitive measure of reach and usefully inform intervention planning and implementation. More research is also needed on the prevalence of sexual difficulties and help-seeking patterns, specifically in ethnic and sexual minorities, to avoid exacerbating existing inequalities in help-seeking.

Younger women with distressing sexual difficulties are more likely than older women to seek help, and both men and women who seek help via the Internet also tend to be younger. Further research is needed to explore this age divide to determine, for example, whether it relates to technology (e.g., access or acceptability) or preferences (e.g., for face-to-face support) or cohort factors (e.g., higher digital literacy in younger generations). This finding has implications for the design and delivery of future IDIs for sexual difficulties.

The findings from the current study can be generalized to the British general population. But given the potential worldwide reach of IDIs, the research needs to be duplicated in other countries, as findings are likely to be dependent on sociocultural contexts.

While the current study demonstrates a potential unmet need for help with sexual difficulties and highlights the potential of IDIs to fill some of the gaps in service provision, more needs to be known about optimal models for delivery, efficacy, mechanisms of action, impact on health inequalities, and cost-effectiveness (Bennett & Glasgow, 2009). Implementation research is needed to explore barriers to dissemination, uptake, and engagement.

## Conclusion

In Britain, more than half of people with distressing sexual difficulties do not seek help for them, which suggests a high level of unmet need in this area. The Internet was commonly reported as a source for seeking help in 2010 to 2012, utilized by 20.7% to 25.6% of those with distressing sexual difficulties. Our data suggest that the Internet is currently underutilized as a source of help for sexual difficulties in Britain—and that, given the level of unmet need, the potential role it could play is significant.
